# The use and reporting of airline passenger data for infectious disease modelling: a systematic review

**DOI:** 10.2807/1560-7917.ES.2019.24.31.1800216

**Published:** 2019-08-01

**Authors:** Margaux Marie Isabelle Meslé, Ian Melvyn Hall, Robert Matthew Christley, Steve Leach, Jonathan Michael Read

**Affiliations:** 1 National Institute for Health Research, Health Protection Research Unit in Emerging and Zoonotic Infections at University of Liverpool, Liverpool, United Kingdom; 2 Institute of Infection and Global Health, University of Liverpool, Liverpool, United Kingdom; 3 School of Mathematics, University of Manchester, Manchester, United Kingdom; 4 Emergency Response Department, Public Health England, Salisbury, United Kingdom; 5 National Institute for Health Research, Health Protection Research Unit in Emergency Preparedness and Response at Kings College London, London, United Kingdom; 6 National Institute for Health Research, Health Protection Research Unit in Modelling Methodology at Imperial College London, London, United Kingdom; 7 Centre for Health Informatics Computation and Statistics, Lancaster Medical School, Lancaster University, Lancaster, United Kingdom

**Keywords:** airline data, mathematical modelling, infectious disease spread, travel, outbreaks, infection, epidemic, passenger data

## Abstract

**Background:**

A variety of airline passenger data sources are used for modelling the international spread of infectious diseases. Questions exist regarding the suitability and validity of these sources.

**Aim:**

We conducted a systematic review to identify the sources of airline passenger data used for these purposes and to assess validation of the data and reproducibility of the methodology.

**Methods:**

Articles matching our search criteria and describing a model of the international spread of human infectious disease, parameterised with airline passenger data, were identified. Information regarding type and source of airline passenger data used was collated and the studies’ reproducibility assessed.

**Results:**

We identified 136 articles. The majority (n = 96) sourced data primarily used by the airline industry. Governmental data sources were used in 30 studies and data published by individual airports in four studies. Validation of passenger data was conducted in only seven studies. No study was found to be fully reproducible, although eight were partially reproducible.

**Limitations:**

By limiting the articles to international spread, articles focussed on within-country transmission even if they used relevant data sources were excluded. Authors were not contacted to clarify their methods. Searches were limited to articles in PubMed, Web of Science and Scopus.

**Conclusion:**

We recommend greater efforts to assess validity and biases of airline passenger data used for modelling studies, particularly when model outputs are to inform national and international public health policies. We also recommend improving reporting standards and more detailed studies on biases in commercial and open-access data to assess their reproducibility.

## Introduction

International movement of individuals through commercial airline travel has been implicated in the transnational dissemination of many infectious diseases and is thought to be the principle mode of human pathogen transfer between continents. Examples include the global dissemination of the outbreak of severe acute respiratory syndrome in 2003 which quickly spread from Hong Kong to North America [1]. The 2009 influenza pandemic [2], which emerged in Mexico and affected more than 208 countries, followed a similar international dissemination [3]. There is, year-on-year, an increasing number of airline travellers, with a total of 1,186 million international tourist arrivals globally in 2015, a 4.6% increase from 2014 and 510 million arrivals more than in 2000 [4]. In addition, tourism visits to emerging economies are now comparable to those of high-income countries, with countries such as Mexico and Thailand entering the top 15 of the most visited destinations. The global trend is expected to keep rising and reach 1.8 billion arrivals in 2030 [4]. Lower fares and greater availability make geographically distant destinations easier to reach for a greater number of people [5]. 

With the volume of airline passengers increasing each year [6], it is important to understand the dynamics of the airline network and its role in disease spread and control [7]. We need to be able to accurately predict international transmission through passenger flow. Mathematical models are useful tools that can estimate the risk of infectious disease importation and exportation by international airline passengers [8], especially in the early stages of an outbreak when accurate reporting may be difficult [9]. Models such as the one developed by Lopez et al. use the force of infection in the visited country to determine the risk to international visitors, assuming an arbitrary number of airline passengers [8]. However, this risk can also extend to new areas when returning passengers carry pathogens back to their country of residence, as was the case in Italy in 2007, when an autochthonous chikungunya outbreak occurred following importation [10]. Mathematical models of pathogen importation/exportation risks usually entail a function of the infection level in the visited country and the airline passenger volume between the two involved geographical locations, as described by Quam and Wilder-Smith [11]. Access to accurate and appropriate data sets describing passenger flow between locations is crucial when developing transmission models of global spread [12]; such models can explore the potential role the airline network may play in the spread of disease, but also predict future spread, particularly when new threats emerge. However, a variety of data sources have been used leading to inconsistency and incomparability between modelling studies [7]. The sources themselves are generally not designed for epidemic modelling purposes. They include data for use within the aviation industry, which may be expensive to access and impose user restrictions, including prohibition to share with a third party [7,12]. Open-access data sources do exist but may be geographically restricted, provide information in forms not easily convertible into passenger numbers or are limited in temporal resolution [7]. 

To gain an overview of the range of airline passenger data sources used by modelling studies, a systematic literature review was designed and conducted. The principal aim of the review was to determine the data types (e.g. passenger numbers and seat capacity) and sources used for the purposes of modelling international infectious disease importation. A secondary aim of the review was to assess the reproducibility of those studies regarding sourcing and use of airline passenger data.

## Methods

### Search strategy

We conducted a search of the literature on 2 October 2017 using PubMed, Web of Science and Scopus with no restriction on the earliest date of the articles returned. A combination of three sets of search terms was used in this review (#1 AND #2 AND #3). The first set (#1) was: ‘air’ OR ‘airline’ OR ‘aviation’ OR ‘flight’ OR ‘airport’ OR ‘passenger’ OR ‘transport*’ OR ‘travel*’ AND NOT ‘pollution’. The second set (#2) was: ‘epidemic’ OR ‘pandemic’. The final set (#3) was: ‘global’ OR ‘international’. The term ‘pollution’ was classed as an exclusionary term as initial scoping suggested that a large proportion of results included pollution studies, which were deemed irrelevant to this review.

We included articles if they matched the following inclusion criteria: (i) they were primary and peer-reviewed research; (ii) they modelled the international spread of human infectious diseases between at least two countries and (iii) the model was parameterised with airline passenger data. We included modelling studies which considered either dynamic models of the transmission process or non-dynamic modelling of the movement of infected individuals. We also permitted the inclusion of any additional articles if they were identified as the source of passenger data used in already selected articles and met the three inclusion criteria above. Although no language restriction was applied to the searches, articles in a language other than English were excluded during the abstract review if no translated version of the abstract could be found. Review articles not containing primary research were also excluded, unless they addressed specifically the use of airline passenger data in epidemic modelling. Articles for which an abstract could not be accessed were excluded at this stage. 

Following deduplication, the full list of abstracts and titles was reviewed and included or excluded by at least two reviewers independently. Any disagreement regarding inclusion of an article in the review was then discussed between all reviewers. The full text of selected articles was accessed and screened for relevance in more detail. Articles for which the full text could not be accessed, which were not open access and could not be accessed through the University of Liverpool or Lancaster University library subscriptions, were excluded. The bibliographies of the selected articles were searched for additional relevant articles, based on title and full text, subject to the same inclusion and exclusion criteria.

### Data collection strategy

From the final selection of articles, we extracted information regarding the airline passenger data used in each article (Table 1). This information focused on the source, type and validity of data used in the study (Table 1, part A) and the reproducibility of data usage judged by pre-defined criteria (Table 1, part B). For the purposes of this review, data validation was defined as the comparison of primary data used in an article against at least one independent and appropriately comparable set of data. An article was deemed to have validated its data source if it cited another independent and comparable data set and contained a comparison between them. To determine reproducibility, each article was assessed for its reporting of data source using the checklist shown in Table 1, part B and scored accordingly. We did not plan or conduct any bias analysis of the selected publications.

**Table 1:** Systematic review on airline passenger data in infectious disease modelling, (A) fields recorded and (B) criteria used to determine reproducibility of articles and sources

Field	Description	Variable	
**A. Data description**			
Article information			
Authors	At least the first three authors, as on article	Text	
Year of publication		Date	
Title		Text	
Publication name		Text	
Data source			
Commercial data	Commercial databases collecting information about flight routings, aircraft size, number of bookings or passengers, e.g. IATA, OAG, Diio	Yes/no	
Tourism surveys	Any surveys done in the context of tourism, e.g. UNWTO	Yes/no	
National passenger surveys	Surveys conducted at airports, e.g. passenger survey	Yes/no	
Airport published information	Data collected and published by airports, may be groups of airports	Yes/no	
Government immigration data	Data collected by governments on migration numbers, inbound passengers	Yes/no	
Other	E.g. information published by airlines		Yes/no	
Unreported or unclear			Yes/no	
Data type				
Seat capacity	Number of seats available on a specific route		Yes/no	
Itinerary	Data include connections, not just information on origin and destination		Yes/no	
Number of flights	Number of flights between cities/airports/countries following a specific routing		Yes/no	
Number of passengers	Data explicitly describe number of passengers travelling		Yes/no	
Tickets sold	Number of tickets sold or booked per routing		Yes/no	
Origin–destination information	Data include origin airport/city/country and destination airport/city/country		Yes/no	
Direct flight information only	Data do not inform on number of passengers taking connecting flights		Yes/no	
Unreported or unclear	Reported information not sufficient to determine data type		Yes/no	
Data time period				
Date range of data is reported			Yes/no	
Date range			Text	
Reporting quality (scoring criteria see Table part B)				
Fully reproducible	All handling and manipulation of the data is described to a detail adequate to enable reproducibility (reproducibility score = 4)		Yes/no	
Partially reproducible	Important information on handling of the data is missing, or methodology is vague (reproducibility score = 3)		Yes/no	
Not reproducible	Information on methods and/or data source is missing and methodology unclear (reproducibility score ≤ 2)		Yes/no	
Data validation				
Data validation attempted	A comparison was made with an independent and appropriate source of information		Yes/no	
Data usage				
Transmission model	Airline passenger information is used to parameterise a model of transmission		Yes/no	
Network analysis	Airline passenger information is described using social network methodology		Yes/no	
Descriptive or illustrative	Airline passenger information is used to illustrate a transmission risk, but no formal analysis or modelling is performed		Yes/no	
Other	None of the above (specify or describe what was done)		Yes/no	
Unclear or unreported	Insufficient information to determine data usage		Yes/no	
Pathogen modelled				
Non-specific	Generic model		Yes/no	
MERS coronavirus			Yes/no	
Seasonal influenza			Yes/no	
Pandemic influenza			Yes/no	
Other (specify)			Text	
**B. Reproducibility^a^**				
Data accessibility (mutually exclusive categories)			Score contribution^b^	
Open source	Publicly available, no restrictions on use, no access fees, and source (where online) still accessible as at January 2017		Yes = +1; No = 0	
Closed source	Publicly available but restricted access, access may be granted following registration and/or fee, e.g. proprietary data		Yes = 0; No = 0	
Not publicly available	Private data, access at discretion of custodian, e.g. airport or airline company information		Yes = 0; No = 0	
Reporting clarity of data source			(All Yes = +1)^c^	
Source identified	The source of the original data is clearly stated		Yes/no	
Data set named	The specific name of the data set or database in the source is reported		Yes/no	
Access date specified	The date(s) on which data were accessed is reported		Yes/no	
Data type reported	The type or unit represented by the data is reported, e.g. number of flights/seats/passengers		Yes/no	
Reporting clarity of data usage				
Data handling reported	Data manipulation before analysis, including data cleaning and/or aggregation, is reported		Yes = +1; No = 0	
Date range of data used				
Data time range reported	The time period covered by the data is reported		Yes = +1; No = 0	
Total reproducibility score	Maximum score = 4. If multiple sources were used in an article, the average score was calculated.			

Diio: data in, intelligence out; IATA: International Air Transport Association; MERS: Middle East respiratory syndrome; OAG: company providing air travel data; UNWTO: World Tourism Organization.

^a^ If studies used a third party’s travel model and if they did not describe the model fully but provide a link or citation, we assessed the cited external documentation for reproducibility.

^b^ Only material using open source data contributes +1 point to the reproducibility score.

^c^ The material must receive a ‘yes’ for all subvariables for this variable to contribute +1 point to the reproducibility score.

## Results

From the 4,012 articles identified in the search, 2,547 were identified as duplicates and rejected, resulting in 1,465 articles which went forward for title and abstract screening (Figure). A further 1,130 were rejected at this stage as they did not meet the inclusion criteria. A total of 335 articles were selected based on their title and abstract and read in full. From these, 223 were rejected: the majority (n = 87) did not contain airline data, 73 were deemed not relevant (did not contain at least two required criteria, such as airline data and model) and 20 used no model. An additional 19 were country-specific, 17 were inaccessible (no access to journal or language barrier), five were reviews and two were not focused on human disease movement. After reading the articles in full, 112 were selected as relevant to this review. Finally, 24 additional articles, not detected by the search but through reading the bibliography of accepted articles, were included after being read in full to determine relevance.

**Figure:**
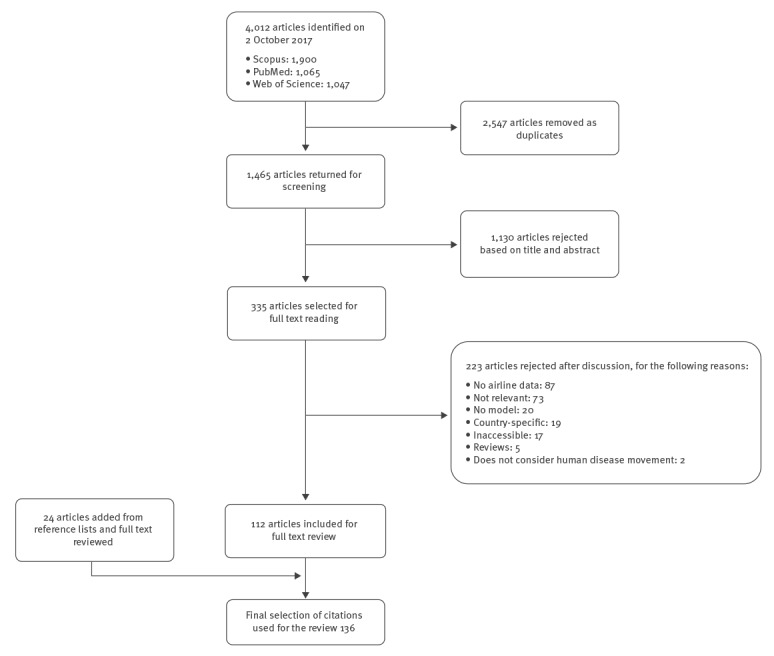
Systematic review on airline passenger data in infectious disease modelling, flow chart of the article selection process

The publication year of the 136 articles selected ranged from 1985 to 2017, with the largest number of articles (n = 17) published in 2016 (Table 2). In the 20 years following the publication by Rvachev and Longini in 1985, the oldest article relevant to this review, only seven relevant articles were published [13-19].

**Table 2:** Systematic review on airline passenger data in infectious disease modelling, list of selected articles with name of data source, information on data validation and reproducibility score (n = 136)

Reference	Sources used	Validation	Reproducibility score^a^
Ajelli et al, 2009 [**22**]	IATA	No	0
Apenteng et al, 2014 [**78**]	Malaysian Department of Statistics	No	2
Apolloni et al, 2013 [**79**]	Airports: Amsterdam, Frankfurt, Gatwick, Hamburg, Hannover, Heathrow, Helsinki, Luton, Munich, Stansted, Teheran, Venice	No	0.33 (0, 0, 1, 0, 1, 0)
Arino et al, 2015 [**80**]	IATA	No	1
Bajardi et al, 2011 [**42**]	IATA	No	0
Balcan et al, 2009 [**21**]	IATA	No	0
Balcan et al, 2010 [**23**]	IATA and OAG	No	0 (0, 0)
Balcan et al, 2009 [**24**]	IATA and OAG	No	0 (0, 0)
Bedford et al, 2015 [**50**]	Civil Aviation Authority	No	3
Bobashev et al, 2008 [**35**]	OAG	No	2
Bogoch et al, 2016 [**81**]	IATA	No	2
Bogoch et al, 2016 [**82**]	IATA	No	2
Bogoch et al, 2015 [**25**]	IATA	No	2
Bowen et al, 2006 [**83**]	OAG (OAG MAX)	No	1
Brannen et al, 2016 [**84**]	US Department of Transportation (Air Carrier Activity Information System)	No	2
Brennan et al, 2013 [**51**]	Twitter	No	3
Brigantic et al, 2009 [**62**]	US Department of Transport	No	1
Brockmann et al, 2013 [**36**]	OAG	No	0
Brockmann et al, 2007 [**85**]	IATA and OAG	No	0 (0, 0)
Brown et al, 2012 [**86**]	Civil Aviation Authorities	No	2
Caley et al, 2007 [**87**]	Unknown	No	0
Carias et al, 2016 [**37**]	OAG	No	2
Cauchemez et al, 2014 [**88**]	IATA	No	1
Chang et al, 2010 [**52**]	Feeyo	No	3
Cheng et al, 2017 [**89**]	ICAO	No	1
Chong et al, 2014 [**90**]	Unknown	No	2
Chong et al, 2012 [**91**]	Hong Kong Tourism Board	No	1
Clements et al, 2010 [**60**]	IATA	No	0
Colizza et al, 2007 [**26**]	IATA	No	0
Colizza et al, 2006 [**27**]	IATA	No	1
Colizza et al, 2006 [**28**]	IATA	No	1
Colizza et al, 2007 [**92**]	IATA	No	0
Colizza et al, 2008 [**29**]	IATA	No	0
Colizza et al, 2007 [**30**]	IATA	No	0
Colizza et al, 2008 [**31**]	IATA	No	0
Cooper et al, 2006 [**93**]	IATA	No	1
Corley et al, 2012 [**64**]	US Department of Transport; OpenFlights.org; OurAirports.com	No	1.33 (2, 1, 1)
**Daniel et al, 2013 [** **20**]	[15,19]	No	0.5 (0.4, 0.6)^b^
Dembele et al, 2017 [**94**]	Unknown	No	0
Dorigatti et al, 2017 [**95**]	UNWTO; Brazilian Ministry of Tourism	No	2.5 (2, 3)
Ekdahl et al, 2005 [**13**]	Swedish Tourist and Travel Database	Yes	3
Epstein et al, 2007 [**96**]	OAG (OAG MAX)	No	0
Flahault et al, 1994 [**14**]	IATA	No	0
Flahault et al, 2006 [**97**]	US Department of Transport; OAG; IATA; ICAO; Back Aviation Solutions; Air Transportation Statistics; Australian International Arrivals; Airbus Industries; Boeing corporation; unknown	No	0.8 (2, 1, 1, 1, 1, 0, 0, 0, 1, 1)
Fraser et al, 2009 [**2**]	OAG	No	2
Gardner et al, 2017 [**98**]	IATA (Passenger Intelligence Services)	No	2
Gardner et al, 2013 [**53**]	IATA	No	3
Gardner et al, 2016 [**99**]	IATA (Air passenger market analysis)	No	2
Gardner et al, 2012 [**54**]	US Department of Transport	No	3
Gardner et al, 2012 [**100**]	US Department of Transport; Eurostat	No	2.5 (3, 2)
Gardner et al, 2015 [**101**]	IATA	No	2
Gautreau et al, 2007 [**102**]	IATA	No	0
Gautreau et al, 2008 [**57**]	IATA	Yes	0
Goedecke et al, 2007 [**103**]	OAG (OAG MAX)	No	2
Gomes et al, 2014 [**66**]	IATA; OAG	No	0 (0, 0)
Gonçalves et al, 2013 [**43**]	IATA; OAG	No	0 (0, 0)
Goubar et al, 2009 [**104**]	ICAO; National Bureau of Statistics of China	No	1 (1, 1)
Grais et al, 2003 [**15**]	US Department of Transport; OAG; IATA; ICAO (Traffic by Flight Stage); Back Aviation Solutions; Air Transportation Statistics; Australian International Arrivals; Airbus Industries; Boeing corporation; unknown	No	0.3 (2, 0, 0, 0, 0, 0, 0, 0, 0, 1)
Grills et al, 2016 [**105**]	Diio	No	1
Hanvoravongchai et al, 2011 [**106**]	Mexican Secretary of communication and transport	No	2
Hatz et al, 2009 [**107**]	UNWTO; UK Office for National Statistics	No	2 (1, 3)
Hollingsworth et al, 2006 [**108**]	Beijing Capital International Airport (Traffic Data); Hong Kong International Airport (Provisional Civil International Air Traffic Statistics); IATA	No	0.67 (1, 1, 0)
Hollingsworth et al, 2007 [**109**]	IATA (International Travel Statistics); Hong Kong International Airport; Beijing Capital Airport	No	0.67 (1, 1, 0)
Hosseini et al, 2010 [**32**]	IATA	No	1
Hsu et al, 2010 [**110**]	Amadeus; Landing.com	No	0.5 (0, 1)
Hufnagel et al, 2004 [**16**]	IATA; OAG	No	0 (0, 0)
Hwang et al, 2012 [**111**]	Diio	No	2
Johansson et al, 2012 [**112**]	OAG (Traffic Analyser); US Department of Transport	No	0.5 (0, 1)
Johansson et al, 2011 [**65**]	OAG (Traffic Analyser); US Department of Transport	No	0.5 (0, 1)
Johansson et al, 2014 [**113**]	Diio	No	2
Kenah et al, 2011 [**114**]	Unknown	No	0
Kernéis et al, 2008 [**115**]	US Department of Transport; OAG; IATA; ICAO; Back Aviation Solutions	No	0.4 (2, 0, 0, 0, 0)
Khan et al, 2009 [**116**]	IATA	No	1
Khan et al, 2014 [**75**]	IATA	No	2
Khan et al, 2013 [**58**]	IATA	Yes	2
Khan et al, 2010 [**48**]	Unknown	No	2
Khan et al, 2012 [**61**]	IATA	No	1
Khan et al, 2010 [**117**]	ACI; Saudi Arabia Authority of Civil Aviation; IATA (Worldwide passenger ticket sales)	No	1 (1, 2, 0)
Khan et al, 2013 [**118**]	IATA	No	2
Knipl et al, 2013 [**119**]	Statistics Canada; unknown	No	1 (1, 1)
Lawyer, 2016 [**120**]	OpenFlights.org	No	2
Lemey et al, 2014 [**38**]	OAG	No	1
**Longini, 1988 [** **17**]	[21]	No	0.6 ^b^
Longini et al, 1986 [**18**]	Air Transport Statistics; Australian International Airport traffic dynamics; ABC World Airways Guide; OAG; ICAO	No	0.4 (0, 1, 0, 0, 1)
Lourenço et al, 2014 [**121**]	Airport: Madeira	No	1
Malone et al, 2009 [**63**]	US Department of Transport	No	1
Marcelino et al, 2009 [**39**]	OAG	No	2
Marcelino et al, 2012 [**122**]	OAG	No	2
Massad et al, 2017 [**123**]	IATA	No	1
Massad et al, 2016 [**124**]	IATA	No	1
Massad et al, 2009 [**125**]	Singapore Tourism Sector Performance	No	2
Massad et al, 2014 [**126**]	Brazilian Ministry of Tourism	No	1
Matrajt et al, 2013 [**127**]	OAG (OAG MAX); unknown	No	1 (2, 0)
Meloni et al, 2011 [**128**]	OAG	No	2
Merler et al, 2010 [**129**]	Eurostat	No	2
Nah et al, 2016 [**130**]	OpenFlights.org	No	2
Nah et al, 2016 [**131**]	OpenFlights.org	No	2
Napoli et al, 2012 [**132**]	CapStat	No	1
Pastore-Piontti et al, 2016 [**44**]	IATA; OAG	No	1 (1, 1)
Paul, et al, 2008 [**133**]	US Department of Transport	No	2
Pinset et al, 2014 [**134**]	UNWTO; UK Office for National Statistics	No	1.5 (2, 1)
Poletto et al, 2016 [**135**]	IATA	No	1
Poletto et al, 2016 [**45**]	IATA	No	0
Poletto et al, 2014 [**136**]	IATA; OAG	No	1 (1, 1)
Poletto et al, 2014 [**33**]	IATA	No	0
Poletto et al, 2012 [**137**]	EuroStat	No	1
Poletto et al, 2013 [**138**]	UK Office for National Statistics	No	1
Polwiang, 2015 [**139**]	Department of Tourism of Thailand	No	2
Quam et al, 2015 [**10**]	IATA	No	0
Quam et al, 2016 [**55**]	Japan National Tourism Organization	No	3
Quam et al, 2016 [**9**]	IATA	No	2
Read et al, 2015 [**77**]	OAG (Traffic Analyser)	No	2
Rocklov et al, 2016 [**140**]	IATA	No	2
Ruan et al, 2006 [**141**]	IATA	No	1
Rvachev et al, 1985 [**19**]	OAG; ICAO; Air Transportation Statistics; Australian International Arrivals; unknown	No	0.6 (1, 1, 0, 1, 0)
Sato et al, 2015 [**142**]	OAG	No	2
Schneider et al, 2011 [**143**]	Unknown	No	0
Semenza et al, 2014 [**74**]	IATA	No	0
Sessions et al, 2013 [**34**]	IATA ; OAG	Yes	2 (2, 2)
Seyler et al, 2009 [**59**]	EuroStat; IATA ; ICAO	Yes	0.33 (1, 0, 0)
Struchiner et al, 2015 [**144**]	Singapore Tourism Board	No	1
Tatem et al, 2006 [**145**]	OAG	No	1
Tatem et al, 2007 [**40**]	OAG (OAG MAX)	No	2
Tatem et al, 2012 [**41**]	US Office of Travel and Tourism Industries; OAG	No	1.5 (2, 1)
Tatem et al, 2006 [**146**]	OAG	No	1
Tian et al, 2017 [**147**]	ICAO	No	2
Tizzoni et al, 2012 [**46**]	IATA; OAG	Yes	0.5 (0, 1)
Tuncer et al, 2014 [**148**]	US Department of Transport	No	2
Urabe et al, 2016 [**149**]	ICAO	No	1
Weinberger et al, 2012 [**56**]	Icelandic Tourism Board; Statistics Iceland; Keflavik Airport	No	3 (4, 3, 2)
Wilder-Smith et al, 2017 [**150**]	UNWTO	No	2
Wilder-Smith et al, 2015 [**151**]	IATA	No	1
Wilder-Smith et al, 2014, [**152**]	IATA	No	2
Wilson et al, 2015 [**153**]	IATA (Airport Intelligence Services – Passenger data)	No	1
Xiao et al, 2015 [**154**]	OAG	No	1
Yoneyama et al, 2012 [**155**]	UNWTO database 1; UNWTO database 2	No	1 (1, 1)

ACI: Airport Council International; Diio: data in, intelligence out; IATA: International Air Transport Association; ICAO: International Civil Aviation Organization; OAG: company providing air travel data; OAG MAX: product produced by OAG; UK: United Kingdom; UNWTO: World Tourism Organization; US: United States.

^a^ Average total score shown, with individual source scores shown in brackets where multiple sources used.

^b^ Where the cited data source was another article, the average score of that article was used.

A wide range of data sources have been used for modelling passenger flow between countries; in total 45 distinct sources were identified (Table 3). Commercial or industry data sources were most often used (14 sources, used in 131 articles), followed by governmental data (14 sources, used in 30 articles). Of the commercial data sources, those most often acknowledged were from the International Air Transport Association (IATA) (61 articles) and OAG, an airline industry company specialising in data provision and analysis (38 articles). Some articles used the airline data directly, however, two articles [17,20] used data from one or more articles (see Table 2) and therefore were also thought of as using industry data. Where a database was named from IATA or OAG sources, OAG MAX was the most common (5 articles). A range of other industry-orientated data sources were cited, including Diio (airline market information), Amadeus (travel reservations database), Feeyo (a Chinese flight scheduler) and OpenFlights.org (an open-access database of flight records contributed by members of the public). Four articles used passenger surveys such as TravelPac from the United Kingdom’s (UK) Office for National Statistics (ONS), and nine articles used tourism surveys (Table 3). Eleven articles used information published by airports, and four other sources were reported (the social media site Twitter, two aircraft manufacturers and EuroStat).

**Table 3:** Systematic review on airline passenger data in infectious disease modelling, data sources identified in the selected articles, grouped by sector (n = 136 articles)

Data source (number of uses; percentage of total uses of any data source)	Number of articles using data source^a^	Reference(s)
**Commercial/industry (n = 131; 62%)**		
International Air Transport Association (IATA)		
IATA − unspecified database	57	[9,10,14-16,20-34,42-46,53,57-61,66,74,75,80-82,85,88,92,93,97,101,102,109,115-118,123,124,135,136,140,141,151,152
IATA − Air passenger market analysis	1	[99]
IATA − Airport intelligence services – passenger data	1	[153]
IATA − International travel statistics	1	[108]
IATA − Passenger intelligence services	1	[98]
OAG (company specialising in airline industry data)		
OAG − Unspecified database	30	[2,15-20,23,24,34-39,41,43,44,46,66,85,97,115,122,128,136,142,145,146,154]
OAG MAX	5	[40,83,96,103,127]
OAG − t 100 database	2	[65,112]
OAG − Traffic analyser	1	[77]
International Civil Aviation Organization (ICAO)		
ICAO − Unspecified database	11	[17-20,59,89,97,104,115,147,149]
ICAO − Traffic by flight stage	1	[15]
Air transport statistics	3	[18-20]
Airports Council International (ACI)	1	[117]
Amadeus	1	[110]
BACK Aviation Solutions Incorporated	4	[15,20,97,115]
CapStat	1	[132]
Diio	3	[105,111,113]
Feeyo	1	[52]
Landings.com	1	[110]
OpenFlights.org	4	[64,120,130,131]
OurAirports.com	1	[64]
**Tourism surveys (n = 9; 4%)**		
Icelandic Tourist Board	1	[56]
Singapore Tourism Board	1	[144]
Turism.se (Swedish tourist and travel commercial database)	1	[13]
World Tourism Organization (UNWTO)	5	[95,107,134,150,155]
United States Office of Travel and Tourism Industries	1	[41]
**National passenger surveys (n = 4; 2%)**		
Brazilian Ministry of Tourism	1	[95]
United Kingdom Office for National Statistics	3	[107,134,138]
**Airport-published information (n = 12; 6%)**		
Amsterdam Airport (Schiphol)	1	[79]
Beijing Capital International Airport	2	[108,109]
German airports (Hannover, Frankfurt, Hamburg, Munich)	1	[79]
Helsinki Airport	1	[79]
Hong Kong International Airport	2	[108,109]
Keflavik Airport	1	[56]
London airports (Heathrow, Gatwick, Stansted, Luton)	1	[79]
Madeira Airport	1	[121]
Teheran Airport	1	[79]
Venice Airport	1	[79]
**Government-published information (n = 33; 15%)**		
United States Department of Transport	14	[15,20,54,62-65,84,97,100,112,115,133,148]
Australian Department of Transport	2	[18,19]
Australian International Airport Traffic	4	[15,18-20]
Brazilian Ministry of Tourism	1	[126]
Department of Tourism of Thailand	1	[139]
Hong Kong Tourism Board	1	[91]
Japan National Tourism Organization	1	[55]
Malaysian Department of Statistics	1	[78]
Mexican Secretary Communication and Transport	1	[106]
National Statistics China	1	[104]
General Authority Of Civil Aviation of Saudi Arabia	1	[117]
Singapore tourism sector performance	1	[125]
Statistics Canada	1	[119]
Statistics Iceland	1	[56]
United Kingdom civil aviation authorities	2	[50,86]
**Other sources (n = 11; 5%)**		
Airbus Industries	3	[15,20,97]
Boeing Corporation	3	[15,20,97]
EuroStat	4	[59,100,129,137]
Twitter	1	[51]
**Unclear or unreported (n = 13; 6 %)**	13	[15,18-20,48,87,90,94,97,114,119,127,143]

^a^ Some articles used more than one data source.

Most data sources contained information about origin and destination (n = 91, 67%) or passenger numbers (n = 73, 54%) (Table 4). Data pertaining to direct flights only were used more often than data pertaining to full passenger itineraries: n=33 and n=27, respectively. Of the 62 studies using IATA as a data source, 15 used information of direct flight only [10,21-34] and of the 38 using OAG, 11 used information of direct flight only [2,23,24,34-41]. Finally, eight articles [21,22,24,42-46] indirectly used IATA data by using the online modelling tool GLEAMviz [47], and two [10,48] by using BioDisapora (now Bluedot.global [49]).

**Table 4:** Systematic review on airline passenger data in infectious disease modelling, frequency of use of each data type identified (n = 136 articles)

Data type^a^	Number of articles using data type		References
	n	%	
Includes information on origin and destination	91	67	[2,9,10,13-16,21,23-26,30,31,34,35,37-42,44,46,48,53-56,58,59,64,66,74,75,77,79,81-83,85,86,88-90,92,93,95,96,98-101,104-107,110-113,116-118,120,122-124,127,130-133,135-141,145-155]
Passenger numbers	74	54	[2,9,10,13-16,25,29,31,34,37,41,45,48,50,53-56,58,59,62-64,74,75,77,79,82,84,86-90,94,95,98-101,104-109,113,116,117,123-126,132-135,137,139-141,144-153,155]
Direct flights only	33	24	[2,10,21-41,54,59,64,79,89,104,111,113,120,137]
Full itinerary	27	20	[10,25,34,53,58,59,61,74,75,81,83,88,98,99,101,112,116,118,123,130,136,140,141,146,152-154]
Unreported or unclear	25	18	[15,17-20,43,51,57,64,65,78,80,91,97,102,109,114,115,119,121,126,127,129,138,143]
Seat capacity	24	18	[21-24,26-28,31,32,35,36,38,40,41,44,46,60,66,93,111,120,122,142,154]
Flight numbers	13	10	[36,39,62,63,83,85,96,103,106,110,127,128,131]
Tickets sold	3	2	[52,64,81]

^a^ An article may have included multiple data types.

According to the set of standards we had established to determine an article’s reproducibility (see Table 1, part B), no article was considered fully reproducible. Eight (6%) articles were deemed partially reproducible (score of 3 or above), where some information regarding the description and use of passenger data was reported [13,50-56]. Of the 45 total data sources identified, 26 were open source, 11 were closed source, and 8 were not publicly available. The date range of the data (start and end date) was reported in 58% (n = 79) studies, and an access date was stated in 25% (n = 34) of the sources used. Data validation as previously defined was performed in 5% (n = 7) of the articles [13,34,46,51,57-59]. Only 40 articles (29%) reported performing any data cleaning or manipulation before using the data set. 

The majority of articles (n = 115; 85%) were concerned with the global spread of infectious diseases, while the analysis of the airline network itself (while modelling pathogen spread) was the next most common purpose (n = 11; 8%). Five articles used passenger data for descriptive or illustrative purposes [13,29,30,60,61], two articles used the data for passenger screening simulations [62,63] and two articles described the development of a public health tool [23,64]. Of the pathogens modelled, pandemic influenza was the most frequent subject of the models (n = 40; 29%) (Table 5). Generic models not focussing on a specific pathogen were also common (n = 23; 17%).

**Table 5:** Systematic review on airline passenger data in infectious disease modelling, pathogens modelled in the selected articles (n = 136)

Pathogen^a^	Number of articles modelling pathogen		References
	n	%	
Generic model (no specific pathogen)	23	17	[20-22,27,28,30,31,35,36,40,48,57,61,65,80,102,103,109,128,137,138,143,149]
Chikungunya virus	6	4	[41,59,75,88,113,132]
*Vibrio cholera*e	1	1	[64]
*Clostridium difficile*	1	1	[60]
Dengue virus	17	13	[10,34,53,55,59,74,89,100,101,121,125,126,132,139,144,147,152]
Ebola virus	7	5	[25,44,66,77,136,142,154]
Hepatitis A virus	1	1	[84]
Human immunodeficiency virus	1	1	[78]
Influenza virus – pandemic	40	29	[2,14-19,23,24,26,32,38,39,42,46,52,54,58,62,63,79,87,90,91,93,96,97,106,108,110,111,115-117,119,120,122,129,148,155]
Influenza virus – seasonal	7	5	[50,51,56,84,114,127,133]
Japanese encephalitis virus	1	1	[107]
*Plasmodium* parasite species	5	4	[41,84,94,134,146]
Measles virus	1	1	[153]
Middle East respiratory syndrome coronavirus	7	5	[33,37,45,99,118,131,135]
Poliovirus	1	1	[151]
Severe acute respiratory syndrome	6	4	[29,83,85,92,104,141]
Smallpox virus	1	1	[43]
*Salmonella enterica* serotypes Typhi and Paratyphi	1	1	[13]*
Vector importation	1	1	[145]
West Nile virus	1	1	[86]
Yellow fever virus	3	2	[95,112,150]
Zika virus	9	7	[9,81,82,98,105,123,124,130,140]

^a^ An article may have included more than one pathogen.

## Discussion

The purpose of this review was to assess the source and usage of airline passenger data used in mathematical models of international infectious disease spread. A total of 136 articles met the inclusion criteria, from which we identified 45 unique data sources.

The majority of these were sources provided on a commercial basis, e.g. IATA, OAG and the International Civil Aviation Organization (ICAO). These commercial sources provide information from the aviation industry for use within that industry and are marketed as being detailed and accurate. The data resolution can be high: for example, passenger data are available stratified by route (including stopovers), fare class, point of origin and time period. There are often restrictions on the use of the data, in particular non-disclosure agreements regarding the data, collection and retrieval methods, and financial charges apply for access [7]. This type of data is essentially closed data: publicly available but with restricted access. Furthermore, the methodology underpinning data collection is generally undisclosed and it is therefore difficult for researchers to assess the quality, representability and biases of the data. Although these data sources may have a number of subsets representing different data types, authors rarely provide more accurate reporting of the data sets, including name of subsets used and date of access, among other criteria. An additional complication is that customers of the same data provider may receive different data depending on the timing, exact parameters of their database query and their subscription levels. 

A number of data sources identified in the review were open-access and include aggregate numbers of passenger published by individual airports, data compiled and released by government agencies (e.g. the UK Office for National Statistics) and information derived from tourism surveys. Although freely available to access, these data sets may not provide the resolution of information required by modelling studies as they typically are limited to passengers departing from or arriving at a specific geographical region or are aggregated over long time periods (annual or quarterly data). In addition, the collection methodology is not always reported for such data sources and there may be biases in the data particularly where reporting is voluntary. Combining information from such sources represents a considerable data challenge.

International travel data describing direct flights only were used more often than those with full itinerary information. Data based on direct flights exclude information on connecting passengers and will therefore underestimate the number of passengers travelling to a specific destination. This limitation is likely to introduce bias, underestimating passenger flow between distant or poorly served locations and overestimating passengers travelling shorter distances [65]. This bias has implications for public health planning as some locations or countries may have an apparent lower risk of importation events because of the lack of direct flights from putative infecting source countries. This may explain the discrepancy during the Ebola epidemic in West Africa in 2014 and 2015, where several studies suggested that the United States (US) was at relatively low risk of importation following the suspension of direct flights. The US did however receive two importations through air travel from the affected area, one was due to a passenger reaching their final destination through indirect flights and the second was a returning healthcare worker [25,66,67].

When considering international travel patterns for public health purposes, accessing information on the number of passengers travelling from an origin to a destination is the most relevant. However, we found that several articles used data for which the unit of measurement was not number of passengers but described passenger traffic in terms of seat capacity – the number of seats on aircraft flying between two specific airports – for which assumptions must be made regarding how full individual flights are and how this may or may not vary with season. In addition, this data type cannot take into account the full routing of a passenger and this information must therefore be inferred from the data or the study needs to state that only direct flights were considered. The variety of data types used for epidemic modelling purposes perhaps reflects the lack of a widely accepted and accessible data source, and this variation in data unit could lead to differences in the conclusions between modelling studies.

To ensure reproducibility by others, studies should report information regarding the source and type of data used, the date of access and any cleaning or manipulation conducted. Our analysis showed that this standard is rarely attained. Reporting the date of access (and date of data extraction if different) is important as several data-providing companies update their data monthly, with retrospective adjustments of values [68]. Few studies reported the date of access to or extraction of the data set. Acknowledging any data cleaning or manipulation is also important for reproducibility [69]: for example, if the authors are considering passengers departing or arriving from cities rather than airports but the data were collected at the airport level, the aggregation of passenger numbers from each airport to the city should be acknowledged by the authors. For additional clarity, it would be useful if the authors reported the stage at which the data was aggregated to city level, whether this was part of the original data, or if this was a data manipulation done by the authors. At the time of writing of this review, there was limited understanding of the sensitivity of this level of data (city level) and how it compares to airport-level data and other aggregated data sets, requiring further analytical work. Overall, the majority of articles were deemed to have methods that were not reproducible, and while eight studies were deemed partially reproducible, none were considered to be fully reproducible. It is incumbent on authors to ensure accurate reporting for all aspects of their methodology; our findings suggest that authors of international disease modelling studies should aim to improve their reporting of source and usage of airline passenger data. We advise authors to reference the fields reported in Table 1, part B, at a minimum, when using any data sets.

Data validation is often required to ensure that the collected data are free from biases and an accurate reflection of the subject or process they describe. For airline passenger data, validation is particularly important if the passenger data are sourced from a commercial company with limited or no collection methodology disclosed. Only seven articles reported validation with at least one independent or appropriately comparable set of observations. While there is no acknowledged gold standard data set, governmental open source data, such as those from the US Department of Transport or Travelpac, do at least have published methodology on which potential biases may be identified.

Many pathogens can be relocated through human movement to populations where susceptibility or a lack of awareness may afford a greater incidence and persistence. Most articles reviewed, where a specific pathogen was considered, investigated transmission or importation of viruses. Only three articles were focused on bacteria (*Vibrio cholera*, *Clostridium difficile* and *Salmonella enterica* serotypes Typhi and Paratyphi), despite the known importance of international travel for the global dissemination of antibacterial resistance [70,71] and the capacity of bacteria to initiate epidemics following importation, e.g. the cholera outbreak on Haiti in 2010 [72]. Pandemic influenza was the disease most often considered by the reviewed articles, which perhaps reflects the global significance of pandemic events and the ease with which pandemic strains have spread historically. The other non-influenza viruses noted in these studies have all initiated outbreaks following introduction through international travel. Outbreaks following introduction occurred in South Korea with MERS Co-V [73], in the Portuguese islands of Madeira (off the coast of Western Africa) with dengue virus [74] and in the Caribbean (leading to imported cases in the US) and Italy with chikungunya virus [75,76]. Finally, the accurate modelling of importation risks for specific pathogens may require very high-resolution passenger data, particularly where routes are indirect and the total travel time from origin to destination is important for screening, taking incubation periods into account [77].

To the best of our knowledge, direct comparisons of commercial with open-access data sets, or between commercial data sets, have not yet been accomplished, preventing an informed decision on which data sets are more suitable to represent airline passengers. Although a direct comparison between commercial data sets is likely to be informative for the modelling community, it is also likely to be expensive. In addition, the presence of a single data set that is agreed by the community to be the best representation of international (and national) airline passenger flow would be ideal, although it may be difficult to realise given proprietorial restrictions of certain data sets. The field should aspire to collaborate with industrial data providers to make accurate passenger data available for research, particularly during global public health emergencies.

### Strengths and limitations of the review

The screening and selection of articles was done in a systematic manner and by two independent reviewers to ensure all relevant articles were included in the selection of articles to be read in full. The full reference lists of accepted articles were read to find additional relevant articles. Although a number of articles were found when going through reference lists, we are confident that this selection was a good representation of the range of airline data used. In addition, no other review that we are aware of is focused on the analysis of the validity and reproducibility of the data used for mathematical models of infectious disease spread by air travel. Limitations of this study include not contacting authors regarding their methods and not including other search engines which may have yielded additional articles but would also have returned a very large number of potential articles to process. In addition, by limiting the articles to international spread only, some articles which focused primarily on spread within a country were excluded, even though they may include relevant data sources.

### Conclusion

We conducted a systematic review to assess the range and reporting of data used by authors to model the international spread of infectious diseases through the airline network. We found 136 articles matching our inclusion criteria and extracted information regarding source, data type, validation assessment and reproducibility. We found a variety of data sources and types used, limited validation performed and poor reporting, rendering many studies unreproducible. We recommend that greater effort is devoted to validation and data sources and that a consensus is achieved on the use of information sources providing airline passenger data. Public health modelling would benefit greatly from the availability of a validated contemporary open-source data source which includes detailed origin–destination information, including connecting passengers, and has high temporal resolution.

## *Note added in proof

During editing following acceptance, the authors became aware of a further four articles that satisfied inclusion criteria but were not discoverable using the search algorithm (De Jong B, Ekdahl K. BMC Public Health. 2006 Dec;6(1):4. Ekdahl K, Andersson Y. BMC Infect Dis. 2004 Dec;4(1):54. Ekdahl K, Andersson Y. Am J Trop Med Hyg. 2005;72(6):825-30. Ekdahl K, Andersson Y. J Infect. 2005;51(3):222-9). The articles all utilised a previously identified data source (Turism.se) and modelled the travel-related risk of campylobacteriosis, giardiasis, salmonellosis and shigellosis infection. This omission affects quantitative elements of Tables 3 to 5, but does not affect our results and conclusions regarding data sources, nor our overall conclusions and recommendations.
